# Human Rights First: Reflections from the Mobilization on Human Rights and Drug Policy Conference

**DOI:** 10.1177/14550725261462713

**Published:** 2026-08-06

**Authors:** Veera Kankainen, Tuukka Tammi

**Affiliations:** 1 Sociology, Centre for Research on Addiction, Control, and Governance, 154260 University of Helsinki Faculty of Social Sciences, Helsinki, Finland; 2 3837 Finnish Institute for Health and Welfare, Helsinki, Finland

**Keywords:** civil society, drug policy, human rights, civic participation, nordic democracy

## Abstract

Nordic countries have traditionally governed drug use and related policies either as criminal or health-related issues – an approach described as a dual-track model. Far less attention has been paid on human rights perspectives in the Nordic drug policy context. Internationally, human rights challenges in drug policies have been increasingly addressed by civil society organizations representing people who use drugs, as well as harm reduction actors. In 2026, the human rights perspective was brought on the Nordic political agenda more prominently than before as, for the first time, the Nordic civil society organizations representing people who use drugs held a joint international conference on the human rights theme. This report provides notes from the conference and discusses current international and Nordic debates on drug policy as a human rights issue.

In Oslo, on 28–29 April 2026, a Norwegian civic organization Association for Humane Drug Policy (Föreningen for Human Narkotikapolitikk, FHR) organized a cross-Nordic confence called Mobilization on Human Rights and Drug Policy. The conference was supported by grant funding that FHR had received from the Nordic Council of Ministers for its project Nordic Mobilization on Human Rights and Drugs, as part of the Council's funding programme DEMOS.

FHR organized the conference in collaboration with seven civil society partners: Brugerforeningen, Gadejuristen, and Brugernes Akademi from Denmark; Humaania päihdepolitiikkaa from Finland; Matthildur Skaðaminnkun from Iceland; ProLAR Nett from Norway; and Linköping Brukarförening from Sweden. Members from all these partner organizations, as well as other civic actors, were invited to the conference.

In addition, the conference brought together human rights advocates, researchers, and policymakers from across the Nordic countries and Europe. We (the authors of this report) participated in the conference as researchers currently studying civic organizations of people who use drugs and their opportunities and challenges to take part in policymaking. We have received a seed funding from the Finnish Foundation of Alcohol Studies for this project.

## The First Day: Redefining Civic Identity of People who use Drugs

The first day of the conference focused on the global goal of shifting drug policies away from criminalization and punitive governance towards a human rights perspective. The day included an impressive range of speakers, panellists, and presentations from across the fields of drug policy and human rights.

Arild Knutsen, a long-term civil society actor and Chair of the Norwegian Association for Humane Drug Policy (FHR), opened the conference. He opposed punitive drug policies and emphasized that people who use drugs should be recognized beyond being targets of social and health care interventions. As he stated, they are neither “criminals” nor “patients,” but human beings with the right to life, freedom, welfare, and dignity.

The United Nations High Commissioner for Human Rights, Volker Türk, gave a video address emphasizing that drug policies should prioritize human rights, public health, and human dignity over punishment and criminalization. He argued that punitive approaches have caused harm and exclusion, and called for evidence-based policies recognizing the rights and participation of people who use drugs.

Kai Spurkland from the Norwegian Institute of Human Rights (NIM) highlighted the relevance of human rights for drug policy, stressing that it should be assessed within broader human rights obligations. Similarly, Damon Barrett from the University of Göteborg reminded that drug policy cannot be separated from international human rights obligations. Drug policies should be assessed according to whether they protect dignity, health, equality, and fundamental freedoms. Although this principle is largely self-evident in most other policy sectors, drug policy has long been treated as an exception, where punitive and coercive measures are more readily accepted.

Ann Fordham, Executive Director of the International Drug Policy Consortium (IDPC), provided a near-historical account of recent developments in drug policy and human rights within the international political community. IDPC is a global network of organizations promoting drug policies based on human rights, public health, and social justice. Drawing on her long career at IDPC, Fordham described how international institutions, including the United Nations, have gradually come to recognize human rights as an integral part of drug policy – and drug policy itself as a human rights issue. She emphasized that this shift has required sustained advocacy by civil society and policymakers over the past two decades.

Neil Woods from Law Enforcement Action Partnership (LEAP) spoke about the economics of drug prohibition. Drawing on his experience as a former undercover police officer, he argued that prohibition creates profitable illicit markets and fuels organized crime and violence.

Jenna-Rose Astwood broadened the discussion by addressing the hidden climate costs of drug prohibition. Her presentation examined the environmental impacts of illicit drug markets and enforcement practices, linking drug policy debates to wider questions of sustainability and environmental justice.

Politicians from across the political spectrum were also present. Anne Linboe, Mayor of Oslo, welcomed the conference participants to Oslo, and Femke Halsema, Mayor of Amsterdam, sent a video greeting. In the afternoon, a panel on youth, policing and drugs featured youth party politicians from Sweden and Norway: Gaute Børstad Skjervø (AUF), Ali Reza Yakhdani (SSU), and Lars Barstad Løvold (FpU), as well as researcher Willy Pedersen (UiO).

## The Second Day: Civil Society Organizations of People who use Drugs

The second day of the conference focused on the current state and future directions of civic activism and advocacy. Anton Basenko, Executive Director of International Network of People Who Use Drugs (INPUD; https://inpud.net), and Leo Jeffreys, Acting Director of European Network of People Who Use Drugs (EuroNPUD; https://www.euronpud.net), introduced their roles and current activities. They highlighted ongoing efforts to strengthen community-led advocacy, defend the rights of people who use drugs, and ensure meaningful involvement of affected communities in drug policy discussions and decision-making.

Peter Sárosi, Executive Director of the Rights Reporter Foundation from Hungary**,** reflected Viktor Orbán's policies undermining human and civil rights. He gave an example of peaceful civic resistance during these policies called the Dance for Freedom, which used dance to express opposition to authoritarianism and to promote humane and evidence-based drug policies.

During the second day, the Nordic civic organizations that had organized the conference introduced their activities and backgrounds. Although differing in size, history, and focus, they shared a commitment to harm reduction, human rights, and improving the social and legal position of people who use drugs.

Three Danish organizations were involved (BrugerForeningen, https://brugerforeningen.dk; Brugernes Akademi, https://brugernesakademi.dk; Gadejuristen, https://www.gadejuristen.dk). Gadejuristen (The Street Lawyers), founded in 1999, provides outreach-based legal aid for socially marginalized people and is known for its work on policing practices and access to rights. Brugernes Akademi (Users’ Academy), established in 2016, focuses on socially marginalized people, peer support, harm reduction, and strengthening the participation among people with lived experience of drug use. Dansk BrugerForening, founded in 1993, is one of the oldest organizations working in this field. Established and led by people who use drugs, the organization advocates for the rights, health, and dignity, and has played a visible role in Danish drug policy debates.

Two Norwegian civic organizations were presented (Foreningen for Human Ruspolitikk, FHR, https://humanruspolitikk.no/en/home; proLAR Nett, https://prolarnett.no). The main organizer, FHR, described its nearly 20-year history in drug policy reform debates, working at local, national, and international levels on harm reduction, user involvement, drug checking services, and improving cooperation between service users and health professionals. Another Norwegian organization, ProLAR Nett, presented its broad work among people receiving opioid substitution treatment (OST). Formed through a merger of two organizations, it works on advocacy, service development, outreach, and peer support.

Other Nordic countries were each represented by one organization (Humaania päihdepolitiikkaa ry, https://hppry.fi; Linköping brukarförening, https://linkopingbrukarforening.se; Matthildur Skaðaminnkun, https://www.matthildurskadaminnkun.is). Finland was represented by Humaania Päihdepolitiikkaa ry (HPP, Humane Drug Policy) which was founded in 2001 and promotes harm reduction, evidence-based drug policy, prevention, and treatment, and advocates a shift “from criminal policy to social policy.” Iceland's Matthildur (founded in 2019) is the first harm reduction organization of the country providing services such as needle and syringe distribution, naloxone access, counseling, and peer-support. Sweden was represented by Linköping Brukarförening,a user-led organization involving family members and people with experiences of dependency. In addition to advocacy work, the organization has been active in campaigns and in creating spaces for dialogue between people who use drugs, relatives, and professionals.

The conference concluded with a draft of future goals presented by John Melhus (FHR) including: (1) ensuring the meaningful societal participation of people who use drugs, their organizations, communities, and allies; (2) integrating harm reduction strategies into national healthcare systems; (3) guaranteeing access to mental healthcare regardless of drug use status; (4) addressing the “Nordic paradox” of restrictive drug policies within otherwise strong welfare states; and (5) ensuring full human rights alignment in all drug policy decisions.

After the end of the official programme, the civic associations held a meeting of their own to discuss these goals and future collaboration. The good news from this meeting reached us researchers on our journey home: for the first time, a Nordic network of organizations advocating for the rights of people who use drugs was established as a direct outcome of the conference.

